# Unusual PDGFRB fusion reveals novel mechanism of kinase activation in Ph-like B-ALL

**DOI:** 10.1038/s41375-023-01843-x

**Published:** 2023-02-21

**Authors:** Teresa Sadras, Fatimah B. Jalud, Hansen J. Kosasih, Christopher R. Horne, Lauren M. Brown, Sam El-Kamand, Charles E. de Bock, Lachlan McAloney, Ashley P. Ng, Nadia M. Davidson, Louise E. A. Ludlow, Alicia Oshlack, Mark J. Cowley, Seong L. Khaw, James M. Murphy, Paul G. Ekert

**Affiliations:** 1 Peter MacCallum Cancer Centre, Parkville, VIC Australia; 2 The Sir Peter MacCallum Department of Oncology, University of Melbourne, Parkville, VIC Australia; 3 Murdoch Children’s Research Institute, Parkville, VIC Australia; 4 Walter and Eliza Hall Institute of Medical Research, Parkville, VIC Australia; 5 Department of Medical Biology, University of Melbourne, Parkville, VIC Australia; 6 Children’s Cancer Institute, Lowy Cancer Research Centre, UNSW Sydney, Kensington, NSW Australia; 7 School of Clinical Medicine, UNSW Medicine & Health, UNSW Sydney, Sydney, NSW Australia; 8 Department of Paediatrics, University of Melbourne, Parkville, VIC Australia; 9 School of Biosciences, University of Melbourne, Parkville, VIC Australia; 10 School of Mathematics and Statistics, University of Melbourne, Parkville, VIC Australia; 11 Children’s Cancer Centre, Royal Children’s Hopsital, Parkville, VIC, Parkville, Australia

**Keywords:** Acute lymphocytic leukaemia, Preclinical research

## To the Editor:

Philadelphia-like (Ph-like) B-cell precursor acute lymphoblastic leaukemia (B-ALL) is a high-risk subtype of leukaemia [1]. It is characterized by a gene expression profile similar to Philadelphia chromosome positive (Ph^+^) ALL, but lacking the *BCR::ABL1* rearrangement. Instead, Ph-like B-ALL represents a heterogeneous subtype driven by a diverse range of genetic aberrations and chromosomal rearrangements that converge on activated tyrosine kinase signalling [2, 3]. Integration of tyrosine kinase inhibitors into treatment regimens of Ph-like ALL patients has demonstrated early evidence of therapeutic efficacy, although results from larger clinical trials are pending [4].

Here we describe a novel PDGFRB lesion in a pediatric patient with Ph-like B-ALL. Unlike other PDGFRB fusions, this *CD74::PDGFRB* variant does not form a chimeric protein, yet is constitutively active and sufficient to drive oncogenic transformation. We provide a model for a unique mechanism of kinase activation in Ph-like ALL which has implications for diagnostic fusion identification and provides key insights into kinase biology.

The patient, a 22-month-old girl, presented with B-ALL and 86% blasts in the bone marrow (BM). Microarray analysis of the BM sample showed losses of chromosome regions 5q32 (partial *PDGFRB* and *CD74*), 7p12.2 (46 kb, intragenic *IKZF1* including exons 4 to 7), and 9p13.3-p13.1 (5 Mb, including *PAX5*, sub-clonal ~70 %). The patient was stratified as high-risk and treated on the COG AALL1131 trial. At the end of induction, residual blasts of 46.3% were still detected. Given indications of a *PDGFRB* lesion, the patient received dasatinib in combination with consolidation chemotherapy, but despite a measurable response, maintained persistent minimal residual disease (0.396%) at the end of consolidation. The patient proceeded to receive a Kymriah CAR T infusion. Additional clinical details are provided in the Supplemental Materials section.

**Fig. 1:**
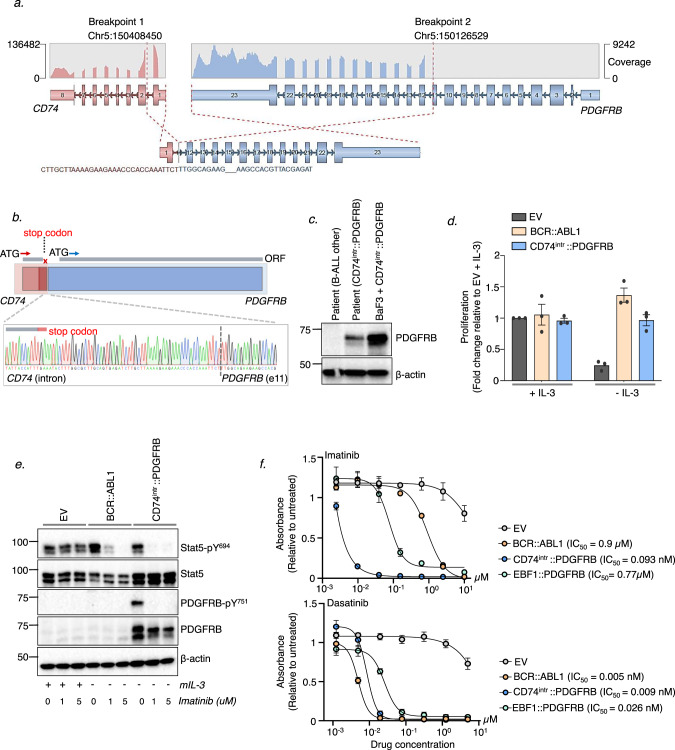
Identification of atypical PDGFRB fusion in Ph-like B-ALL patient. **a** RNA-sequencing analysis identified a novel *CD74::PDGFRB* fusion between *CD74* (exon 1) and *PDGFRB* (exon 11) on chromosome 5. Image shown is modified from a fusion visualization tool Arriba [12]. RNA read coverage is shown across the genes involved in the fusion. **b** Sanger sequencing of the breakpoint shows a retained CD74 intron sequence of 75 bp resulting in a stop codon before the fusion junction. A putative alternative open reading frame (ORF) starting at an ATG within PDGFRB exon 12 is also indicated. **c** Western blot analysis of PDGFRB in bone marrow MNCs from CD74^intr^::PDGFRB^+^ patient. Ba/F3 cells expressing CD74^intr^::PDGFRB and a second B-ALL patient with no known PDGFRB lesion were used as controls. **d** Proliferation of BCR::ABL1 and CD74^intr^::PDGFRB expressing Ba/F3 cells following 48-hour IL-3 withdrawal. Proliferation was measured by luminescence relative to the EV control in IL-3 (day 0), using the CellTiter-Glo® 2.0 reagent. Data shows Mean ± SEM (*n* = 3). **e** Western blot analysis of BCR::ABL1 and CD74^intr^::PDGFRB expressing Ba/F3 cells treated with 0, 1 or 5 μM imatinib for 6 hours. **f** Viability of BCR::ABL1, CD74^intr^::PDGFRB and EBF1::PDGFRB expressing Ba/F3 cells treated with a dose titration of imatinib (top panel) or dasatinib (lower panel) for 72 hours, measured using the CellTiter-Glo® 2.0 reagent. Data is normalized to untreated cells. IC_50_ values for each cell line is indicated. Data shows Mean ± SEM (*n* = 3).

We performed RNA-sequencing on the diagnostic sample. Gene expression analysis identified a Ph-like signature [5], and highlighted aberrant expression of *PDGFRB* (Supplementary Fig. 1a). Sequence analysis confirmed a ~240 kb interstitial deletion in chromosome 5, resulting in a fusion between *CD74* (exon 1) and *PDGFRB* (exon 11) (Fig. 1a). The fusion was validated by PCR amplification of the breakpoint (Supplementary Fig. 1b), and the full length *CD74::PDGFRB* cDNA was amplified and cloned into an MSCV-IRES-GFP (MIG) expression plasmid. Sanger sequencing revealed a retained 75 bp *CD74* intronic sequence resulting in a stop codon before the fusion junction (Fig. 1b). We henceforth refer to this novel fusion as CD74^intr^::PDGFRB. A previous report described a *CD74*::*PDGFRB* variant resulting in an in-frame fusion linking *CD74* exon 6 and *PDGFRB* exon 11 [6]. However, our patient’s fusion is distinguished by the absence of a clear open reading frame (ORF) to drive translation of an in-frame fusion protein. To determine whether a PDGFRB species is produced as a result of the CD74^intr^::PDGFRB fusion, we used western blotting to analyze leukemic mononuclear (MNC) cells from the patient (Fig. 1c). Strikingly, an immunoreactive PDGFRB species of ~60 kDa was detected in the patient MNCs corresponding to an equivalent product detected in murine Ba/F3 cells engineered to express the *CD74*^*intr*^*::PDGFRB* cDNA cloned directly from the patient.

To functionally characterize the novel *CD74*^*intr*^*::PDGFRB* fusion, we stably transduced IL-3-dependent mouse pro-B Ba/F3 cells with *CD74*^*intr*^*::PDGFRB-*MIG or an empty vector (EV) control. *BCR::ABL1* was used as a positive control known to confer cytokine-independent growth of Ba/F3 cells. In the absence of IL-3, expression of CD74^intr^::PDGFRB was sufficient to drive cytokine independent proliferation similar to BCR::ABL1 (Fig. 1d). Consistent with constitutive activation of PDGFRB, transformed CD74^intr^::PDGFRB^+^ Ba/F3 cells grown in the absence of IL-3 showed phosphorylation of PDGFRB (Y751) and Stat5 (Y694) that was blocked by treatment with imatinib (Fig. 1e). These findings were recapitulated in primary murine IL-7 dependent pre-B-cells transduced with *CD74*^*intr*^*::PDGFRB* (Supplementary Fig. 1c). Importantly, CD74^intr^::PDGFRB-expressing Ba/F3 cells were highly sensitive to both imatinib and dasatinib in vitro (Fig. 1f).

**Fig. 2:**
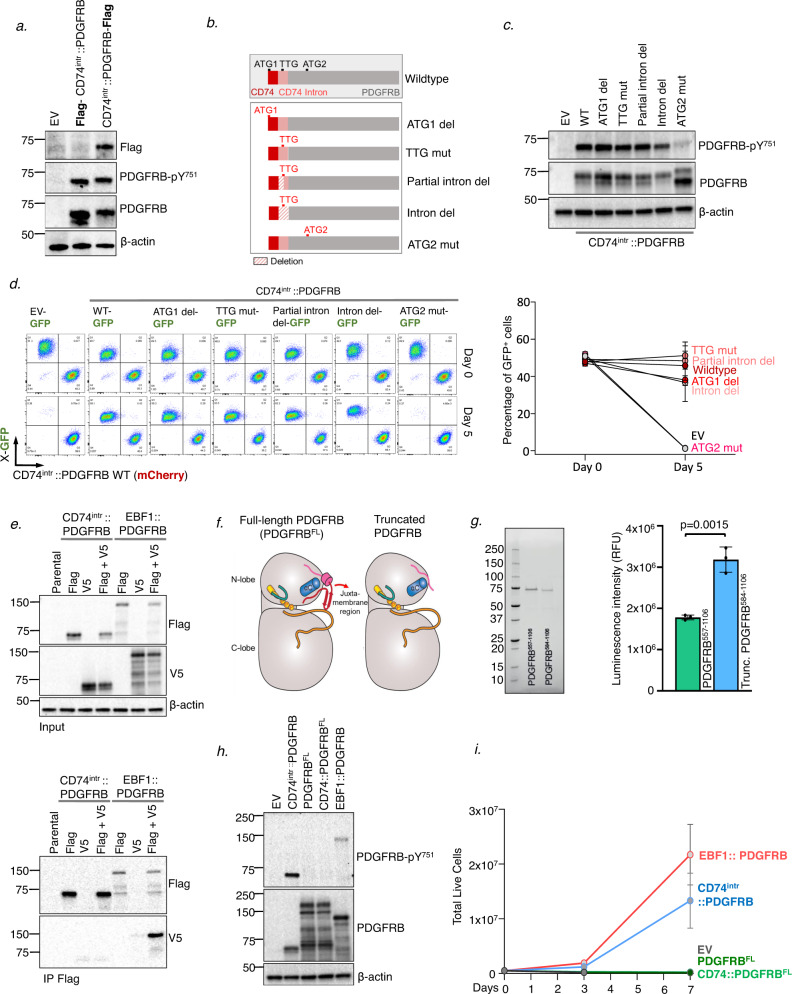
CD74^intr^::PDGFRB is driven by a non-canonical translation start site. **a** N- and C-terminal FLAG tagged CD74^intr^::PDGFRB constructs were transduced into Ba/F3 cells and analysed by Western blotting. **b** Schematic diagram of CD74^intr^::PDGFRB constructs with start codon mutations or deletions, and partial and full intron deletion. **c** Western blot analysis of CD74^intr^::PDGFRB mutants depicted in **b**, expressed in Ba/F3 cells. **d** Growth competition of Ba/F3 lines transduced with CD74^intr^::PDGFRB mutants. Ba/F3 lines expressing EV or CD74^intr^::PDGFRB constructs in GFP-expression plasmid, were mixed 1:1 with Ba/F3 cells expressing CD74^intr^::PDGFRB in mCherry-expression plasmid. GFP and mCherry populations were measured at day 0, and 5 days after mIL-3 withdrawal. Left panel shows representative FACS data, and right panel shows quantified GFP^+^ percentages (Mean ± SEM) from 3 independent experiments. **e** FLAG-co-immunoprecipitation in HEK293T cells expressing FLAG or V5-tagged CD74^intr^::PDGFRB constructs, alone, or in combination. EBF1::PDGFRB was used as a positive control. Shown is whole cell lysate input (top panel) and western blot analysis of FLAG-immunoprecipitate elutes probed for FLAG or V5 (bottom panel). **f** AlphaFold2 structural modelling of full length PDGFRB (PDGFRB^FL^) and CD74^intr^::PDGFRB highlighting loss of juxtamembrane region in CD74^intr^::PDGFRB. **g** Left Panel: Reducing SDS-PAGE analysis of purified PDGFRB visualized by stain-free imaging to illustrate the purity of each PDGFRB preparation. Right Panel: In vitro kinase activities of PDGFRB^557–1106^ and truncated PDGFRB^584–1106^ encoded by the CD74^intr^::PDGFRB fusion. Individual data points are plotted; the bar and error bars shown represent mean ± SD of three independent ADP-Glo assays. Presented data are representative of three technical replicates (*n* = 9). Statistical significance was calculated using an unpaired Student’s *t* test. **h** Western blot analysis of Ba/F3 lines expressing PDGFRB variants. **i** Proliferation of Ba/F3 cells following withdrawal of IL-3 measured by trypan blue exclusion. Data shows Mean ± SEM (*n* = 3). **h** Western blot analysis of Ba/F3 lines expressing PDGFRB variants. **i** Proliferation of Ba/F3 cells following withdrawal of IL-3 measured by trypan blue exclusion. Data shows mean ± SEM (*n* = 3).

Given the unusual structure of the *CD74*^*intr*^*::PDGFRB* sequence, and the apparent lack of an ORF starting at the canonical ATG of CD74 exon 1, we investigated whether translation may be driven by an alternate start site. For this, N- and C-terminal FLAG-tagged versions of *CD74*^*intr*^*::PDGFRB* were expressed in Ba/F3 cells. While equivalent levels of p-PDGFRB (Y751) and total PDGFRB were detected in cells expressing the N- or C-terminal FLAG constructs (Fig. 2a), the FLAG epitope was only detected in cells expressing the C-terminal tag. This strongly suggests that translation of PDGFRB protein is not driven from the first ATG (ATG-1) within CD74. To validate this, and to more stringently dissect whether translation is driven from elsewhere within the CD74-portion, we generated a construct with deletion of the ATG-1 codon from the *CD74*^*intr*^*::PDGFRB* sequence. We also sequentially deleted or mutated putative translation start sites within the *CD74*^*intr*^*::PDGFRB* fusion including a non-canonical TTG-codon within the retained *CD74*-intronic region, and a putative ATG start site (ATG-2) in exon 12 of *PDGFRB* (Fig. 2b). These constructs were expressed in Ba/F3 cells, and levels of p-PDGFRB (Y751) and PDGFRB were measured by western blotting (Fig. 2c). This revealed that only mutation of ATG-2 disrupted expression of an active protein. Strikingly, growth competition analyses of Ba/F3 lines expressing the CD74^intr^::PDGFRB mutants (in GFP expression plasmids), competing with WT CD74^intr^::PDGFRB (in mCherry expression plasmid) confirmed that mutation of ATG-2 abolished the transforming potential of the CD74^intr^::PDGFRB fusion (Fig. 2d).

Together, these results show that protein translation does not commence within *CD74*, and that the product from the *CD74*^*intr*^*::PDGFRB* fusion likely arises from an internal ATG within *PDGFRB* itself, encoding a truncated PDGFRB molecule. In an analogous approach, we assessed the capacity for the *CD74*-region to promote protein translation by generating an artificial hybrid construct of *CD74* with the kinase domain of *ABL1*. This confirmed that the *CD74*-sequence from *CD74*^*intr*^*::PDGFRB* is unlikely to drive translation of a fusion protein (Supplementary Fig. 1d).

Our findings provide evidence for a remarkable scenario, in which aberrant expression of a ‘loose’ PDGFRB kinase domain in B-cell progenitors is sufficient to drive auto-activation and leukemic transformation. This is particularly unique in the context of Ph-like ALL, which is characterized by expression of chimeric fusion proteins in which tyrosine kinase domains are fused in-frame to distinct N-terminal partners (commonly lymphoid transcription factors). Constitutive kinase activation of oncogenic chimeric-fusions is driven by retained oligomerization domains of N-terminal partner proteins, which mediate fusion homodimerization and subsequent auto-phosphorylation of the kinase domains. Notably, oligomerization domains of the partner proteins are indispensable for constitutive activation of other PDGFRB fusions (e.g. ETV6::PDGFRB and EBF1::PDGFRB [7]). Interestingly, co-immunoprecipitation (coIP) of FLAG-tagged CD74^intr^::PDGFRB in HEK293T cells suggests that unlike EBF1::PDGFRB, CD74^intr^::PDGFRB does not strongly dimerize (Fig. 2e). However, this does not exclude the possibility of a weak or transient dimerization driven instead by the stoichiometry of the abundance of the fusion. We note, for instance, that recombinant truncated PDGFRB (PDGFRB^584–1106^) expressed and purified from insect cells elutes at a molecular weight consistent with a dimer (Supplementary Fig. 2a). Nonetheless, these findings suggest an alternative mechanism of activation for CD74^intr^::PDGFRB which may bypass the requirement for dimerization domains in the driver fusion.

To understand how a truncated PDGFRB kinase domain may be constitutively activated, we performed AlphaFold2 structural modelling comparing the intracellular portion of full-length (FL) human PDGFRB (PDGFRB^FL^) and CD74^intr^::PDGFRB (Fig. 2f, Supplementary Fig. 2b). This revealed that the juxtamembrane (JM) domain partially occupies the ATP binding site by running parallel with the αC helix, before forming a β-strand hairpin that abuts the C-lobe of the kinase domain, occluding the active site. This is consistent with the experimental structure of the related protein, PDGFRA, where the active site is analogously occluded by the JM region [8]. In contrast, the truncated form of PDGFRB, encoded by the CD74^intr^::PDGFRB fusion, lacks the JM region and models predict that the active site is not obstructed in this setting. To test this hypothesis, we generated recombinant proteins to measure the kinase activity of PDGFRB^557–1106^ relative to the truncated form that arises from CD74 fusion, PDGFRB^584–1106^, in an ADP-Glo assay with a Poly-(Glu,Tyr) peptide substrate. While the replete intracellular portion of PDGFRB (PDGFRB^557–1106^) exhibited some catalytic activity, the truncated PDGFRB^584–1106^ protein displayed approximately double in vitro kinase activity (Fig. 2g).

Hence, we hypothesized, that unlike the ‘truncated’ CD74^intr^::PDGFRB protein, expression of the full-length PDGFRB (PDGFRB^FL^) receptor may be ineffective in driving transformation as it is locked in an inactive state. To test this idea, we generated Ba/F3 lines expressing either the *CD74*^*intr*^*::PDGFRB* fusion or *PDGFRB*^FL^. We also included a hybrid between *CD74* and *PDGFRB*^FL^ (CD74::PDGFRB^FL^), fusing the *CD74* region to *PDGFRB* exon 1. Immunofluorescence staining revealed that both CD74^intr^::PDGFRB and PDGFRB^FL^ are dispersed through the cell cytoplasm (Supplementary Fig. 2c). Strikingly however, active PDGFRB protein (assessed by Y751 phosphorylation) was only observed in Ba/F3 cells expressing the CD74^intr^::PDGFRB construct, or EBF1::PDGFRB (Fig. 2h). Moreover, while Ba/F3 cells expressing CD74^intr^::PDGFRB proliferated in the absence of IL-3, cells expressing PDGFRB^FL^ or the CD74::PDGFRB^FL^ hybrid did not (Fig. 2i, Supplementary Fig. 2d).

It remains to be determined whether there are other examples of cancer driven by a non-chimeric truncated PDGFRB protein or whether this mechanism of oncogenic activation extends to other kinases. Nonetheless, our observations are broadly relevant to oncogenic signaling given the recognized autoinhibitory function of the juxtamembrane domain in other type III receptor tyrosine kinases including PDGFRB/A [8, 9], c-KIT [10] and CSF1R [11]. Interestingly, molecular dissection of the *FIP1L1::PDGFRA* fusion, which occurs in chronic eosinophilic leukemia, demonstrated that FIP1L1 is entirely dispensable for PDGFRA activation, and that instead it is the partial loss of the JM domain within PDGFRA that drives constitutive activation of the fusion [9]. In the setting of PDGFR fusions which retain the intact JM domains (e.g. ETV6::PDGFRA [9], EBF1::PDGFRB [7]), autoinhibition is instead overcome by enforced dimerization driven by the N-terminal fusion partner, representing two distinct mechanisms by which PDGFR-family kinases may become constitutively activated in malignancy.

We present a unique example of aberrant expression of a non-chimeric ‘loose kinase domain’, which is targetable by ABL-class inhibitors (e.g. imatinib), driving Ph-like B-ALL. Our findings have critical implications for current pipelines of sequencing-based fusion identification, in which putative cancer drivers may be disregarded following out-of-frame predictions. This work also highlights the merit of functional validation of structurally unusual lesions, which may ultimately provide an invaluable opportunity for administration of targeted compounds in treatment regimens.

## Supplementary information

Supplementary Materials, Methods and Figures

## Data Availability

All data generated or analysed during this study are included in this published article and its Supplementary information files. Any other data may be requested from the corresponding author
