# Supplementary material for: Unusual PDGFRB fusion reveals novel mechanism of kinase activation in Ph-like B-ALL
Source: Leukemia. 2023 Feb 21;37(4):905–9. doi: 10.1038/s41375-023-01843-x (PMC10079538; doi:10.1038/s41375-023-01843-x)

Supplementary Material

Unusual PDGFRB fusion reveals novel mechanism of kinase activation in Ph-like B-ALL

Teresa Sadras, Fatimah B. Jalud, Hansen J. Kosasih, Christopher R. Horne, Lauren M. Brown, Sam El-Kamand, Charles E. de Bock, Lachlan McAloney, Ashley P. Ng, Nadia M. Davidson, Louise E. A. Ludlow, Alicia Oshlack, Mark J Cowley, Seong L Khaw, James M. Murphy, Paul G. Ekert.

Materials and Methods

Comprehensive case description of patient

The patient, a 22-month-old girl, presented with a short history of fever, cervical lymphadenopathy and hyperleukocytosis, with initial white cell count of 410 x 10e9/L. She was diagnosed with precursor B cell acute lymphoblastic leukemia with 86% blasts in the bone marrow, and low level CNS involvement (CNS2a). Based on NCI criteria, the patient was stratified as high risk and treated following the COG AALL1131 four drug induction protocol (vincristine 1.5mg/m^2 days 1, 8, 15, 22; dexamethasone 10mg/m^2/day days 1-14; daunorubicin 25mg/m^2 days 1, 8, 15, 22; PEG-asparaginase 2500 units/m^2 day 4; intrathecal cytarabine day 1; intrathecal methotrexate days 8 and 29), but demonstrated a poor early response, with peripheral blood minimal residual disease by flow cytometry of 27% on day eight of induction. Microarray analysis of the bone marrow sample showed relative losses of chromosome regions 5q32 (partial *PDGFRB* and *CD74*), 7p12.2 (46kb, intragenic *IKZF1* including exons 4 to 7), and 9p13.3-p13.1 (5Mb, including *PAX5*, sub-clonal ~70 %), with *PDGFRB* rearrangement subsequently confirmed on fluorescence in situ hybridization (FISH) assay. At the end of the induction, residual blasts of 46.3% were detected, consistent with induction failure. Given the presence of a *PDGFRB* fusion gene mutation, the patient was commenced on dasatinib 60mg/m^2 daily in combination with ongoing protocol chemotherapy as per AALL1131 (2 consolidation cycles, each comprising cyclophosphamide 1000mg/m^2 day 1, cytarabine 75mg/m^2 days 1-4 and 8-11, 6-mercaptopurine 60mg/m^2 days 1-14, then vincristine 1.5mg/m^2 days 15 and 22, PEG-asparaginase 2500 units/m^2 day 15, with four intrathecal methotrexate injections weekly in the first cycle; then interim maintenance comprising high dose methotrexate 5g/m^2 days 1, 15, 29 and 43, and 6-mercaptopurine 25mg/m^2 days 1-56, with intrathecal methotrexate on days 1 and 29) , but despite improved depth of cytoreduction, demonstrated a persistent positive bone marrow minimal residual disease (MRD) (0.396%) at the end of consolidation, and 0.410% at mid-interim maintenance. The patient proceeded to receive tisagenlecleucel CAR T infusion. While MRD post CAR T therapy was negative by flow cytometry at day 30, 60 and 100, it was persistently detectable in the 10^{-4} range by PCR (using *IKZF1* deletion and *TCRD* molecular markers). In this context, and with evidence suggestive of early loss of CAR T cell persistence at day 100, the patient subsequently proceeded to TCR alpha-beta depleted parental donor haploidentical haematopoietic stem cell transplant (HSCT), with chemotherapy-only conditioning (busulfan, fludarabine, thiotepa) without total body irradiation due to young age. Bone marrow assessment at day 30 post-

HSCT demonstrated negative MRD by flow cytometry, but positive molecular MRD at $<1x10^{-4}$ and $<5x10^{-4}$ on *TCRD* and *IKZF1* deletion markers respectively. The patient proceeded to receive four donor lymphocyte infusions together with cyclosporin, with no evidence of graft versus host disease. Subsequent MRD assessments up to two years post-HSCT have been negative, and the patient remains in remission 30 months post-HSCT.

RNA-sequencing of patient material

RNA was extracted from bone marrow mononuclear cells of the patient using the AllPrep DNA/RNA/miRNA Universal Kit (Qiagen). After quality control, high quality RNA was subjected to library preparation using TruSeq Stranded Total RNA Ribo-Zero Gold (Illumina). The library was sequenced on the Illumina NovaSeq 6000 at a sequencing depth of 80M reads using 2x150bp paired-end reads. The .fastq files were aligned to a human reference genome (GRCh38) using STAR[1] after trimming the adapter sequences using Trimmomatic[2]. The resulting .bam file was run on Arriba[3] using the suggested parameters for fusion detection.

Detection and amplification of $CD74^{intr}$::PDGFRB Fusion

The *$CD74^{intr}$::PDGFRB* fusion transcript was identified by cDNA PCR from the case patient using the following primers sets (1) (breakpoint) CD74e1F1: ATGCACAGGAGGAGAA GCAGGAGC; PDGFRBe13R2: TTTCATCGTGGCCTGAGAATGGCTC (2) (breakpoint) CD74e1F2: ATCAGAAGCCAGTCATGGATGACCAG; PDGFRBe13R2: TTTCATCGTG GCCTGAGAATGGCTC (3) (full-length) CD74e1F1: ATGCACAGGAGGAGAAG CAGGAGC; PDGFRB_FL_R1: CTACAGGAAGCTATCCTCTGCTTCCGCC. PCR products were purified using NucleoSpin® Gel and PCR Clean-Up (Takara) and further validated through Sanger sequencng. The full length *$CD74^{intr}$::PDGFRB* fusion transcript was cloned into an MSCV-IRES-GFP expression plasmid using In-Fusion® Snap Assembly (Takara).

Cell culture

Ba/F3 cells were maintained in RPMI 1640 medium (ThermoFisher) supplemented with 10% FBS and 10ng/mL recombinant murine interleukin-3 (PeproTech). Human embryonic kidney 293T (HEK293T) cells were maintained in in DMEM (ThermoFisher) supplemented with 10% FBS. All cell cultures were maintained in incubator at 37 °C with 5% CO_2, and confirmed mycoplasma negative.

Murine pre-B cell culture

Primary murine pre-B cultures were established from C57BL/6 mouse bone marrow (BM) as previously described[4]. For this, bone marrow cells were harvested from ~10-week-old mice by flushing cavities of femur and tibia with cold media and cells were filtered through a 70µm filter to generate single cell suspensions. Bone marrow cells were then cultured in IMDM GlutaMAX™ (ThermoFisher) supplemented with 20% FBS, 55µM 2-ME and 10ng/ml of murine IL-7 (mIL-7) (PeproTech). Cells were maintained in presence of mIL-7 for at least 7 days to generate IL-7 dependent pre-B cells. To produce CD74^{intr}::PDGFRB lines, pre-B cells were retrovirally transduced with CD74^{intr}::PDGFRB and then mIL-7 was removed to promote the outgrowth of transformed cells.

Retroviral transduction

Viral supernatant was produced by transfecting 4 x 10^6 HEK293T cells plated overnight in T75 flasks with Lipofectamine™ 2000 Transfection Reagent (ThermoFisher), containing 10µg purified plasmid DNA, 10µg pHIT60 (gag-pol) and 2.5µg pHIT123 (ecotropic env). The packaging plasmids were a kind gift from Markus Müschen. Viral supernatant was collected and filtered through a 0.45 µm filter, loaded into RetroNectin® (Takara) coated non-tissue culture treated plates and spun for 60 min at 2000 x*g*. Viral supernatant was discarded and 3 x 10^6 Ba/F3 or pre-B cells were loaded onto the plates and spun for 30 min at 600 x*g*. Fluorescence activated cell sorting (FACS) was used to purify *GFP* positive cell populations 72 hours post-transduction using the BD FACSAria Fusion Flow Cytometer.

Proliferation and viability assays

Proliferation of Ba/F3 lines was measured using CellTiter-Glo® Luminescent Cell Viability Assay following manufactures instructions (Promega). For long-term viability experiments in the absence of IL-3, Ba/F3 cells were washed 3x in PBS, and seeded at a concentration of 500,000 cells/mL in 1mL RPMI+10%FBS (v/v) on a 24-well plate. The cells were counted on day 3 and day 7 using Trypan Blue.

Growth Competition experiments

Growth competition of CD74^{intr}::PDGFRB mutants:

Ba/F3 cells containing CD74^{intr}::PDGFRB mutants or empty vector (expressing GFP) were mixed 1:1 with Ba/F3 cells expressing CD74^{intr}::PDGFRB WT (expressing mCherry). GFP and mCherry percentages at 0 and 5 days after IL-3 withdrawal were measured by flow cytometry.

Growth competition on PDGFRB-expressing Ba/F3 lines in presence of imatinib:
Ba/F3 cells containing fusions or empty vector (expressing GFP) were mixed 1:1 with parental Ba/F3 cells to a total number of 500,000 cells in 1mL RPMI+10% FBS ± 1µM Imatinib on a 24-well plate. Total GFP % was monitored by FACS and the cells were counted every day for 4 days with an additional data collected on day 7. For analysis, the GFP % obtained from FACS was used to determine the number of GFP cells and non-GFP cells by extrapolating the values to a total number of live cells collected during cell counting with trypan blue.

Immunofluorescence

Ba/F3 cells ($0.5x10^6$ cells) were seeded onto Nunc Laboratory-TekII chambers in serum-free RPMI and incubated at 37°C/5%CO^2 for 1 hour. The cells were quickly rinsed with PBS, then fixed in 4% v/v Paraformaldehyde in PBS for 15 minutes at room temperature (RT). After a quick wash with PBS, the cells were permeabilised in chilled methanol for 5 minutes at -20°C. Cells were blocked with 5% v/v goat serum in PBS + 0.3% Triton X-100 for 1 hour at RT. The cells were then stained with total-PDGFRB antibody (1/100) (CST# #3169) in PBS containing 1% Bovine Serum Albumin (BSA) and 0.3% v/v Triton X-100 (antibody dilution buffer) overnight at 4°C. After washing the cells with PBS, they were incubated with goat anti-rabbit Alexa Fluor 647 antibody (1/200) (Life Technologies) in antibody dilution buffer for 2 hours at RT. After final washes in PBS, cells were preserved in VECTASHIELD Antifade Mounting Medium with DAPI (Vector Laboratories). The cells were imaged using Zeiss LSM 780 confocal microscope at 40x magnification with scaling (per pixel) of 0.07µm x 0.07µm and an image size (pixels) of 2048 x 2048.

Western blot analysis

Cells were lysed with CelLytic Buffer (Sigma), containing PhosSTOP™ (Sigma) and cOmplete™, Mini, EDTA-free Protease Inhibitor Cocktail (Sigma) according to the manufacturer's protocol. Samples were loaded on 4–15% Criterion™ TGX Stain-Free™ Protein Gels with Tris/Glycine/SDS electrophoresis buffer (25 mM Tris, 192 mM glycine, 0.1% SDS, pH 8.3; Bio-Rad). Protein gels were transferred using the Trans-Blot Turbo RTA Midi 0.2 µm Nitrocellulose Transfer Kit and the Trans-Blot® Turbo™ Transfer System (Bio-Rad). The following primary antibodies were used: phospho-Y694 Stat5 (CST# 4322), Stat5 (CST# 94205), phospho-Y751 PDGF receptor β (CST# #3161), PDGF receptor β (CST# #3169), and c-Abl (CST #2862), Monoclonal Anti-FLAG M2 (Sigma-Aldrich #F1804) and β-actin (CST #58169). Membranes were then incubated with alkaline-phosphatase conjugated

secondary antibodies (Invitrogen). Proteins were detected using CDP-Star™ Substrate (ThermoScientific). Images were acquired and analysed using ChemiDoc XRS+ System (BioRad) and Image Lab.

Co-Immunoprecipitation

HEK293T (4.0 x 10^6 cells) were plated overnight in a T75 flask and transfected with Lipofectamine™ 2000 Transfection Reagent (ThermoFisher). HEK293T cells were transfected with purified plasmid DNA from FLAG (in MSCV-GFP) or V5 (in MSCV-mCherry) tagged $CD74^{intr}$::PDGFRB or EBF1::PDGFRB constructs alone or in combination. Transfection efficiency was confirmed by imaging. Cells were maintained in DMEM supplemented with 10% FBS and lysed 72 hrs post transfection with CelLytic™ M Buffer (Sigma), containing PhosSTOP™ (Sigma) and cOmplete™, Mini, EDTA-free Protease Inhibitor Cocktail (Sigma), prepared according to the manufacturer's protocol. Co-Immunoprecipitation of FLAG tagged protein was done using 1 mg/mL lysate and Pierce Anti-DYKDDDDK Magnetic Agarose (ThermoFisher) according to the manufacturer's protocol. FLAG tagged proteins were eluted with 60 uL of 1.5mg/mL Pierce™ 3x DYKDDDDK Peptide (ThermoFisher). Samples were analysed by western blotting using Monoclonal Anti-FLAG M2 (Sigma-Aldrich #F1804), V5-tag (CST#13202) and β-actin (CST #58169) primary antibodies.

Protein Modelling

AlphaFold2 models of human PDGFRB were generated using an advanced MMseq2 and HHsearch pipeline with Colabfold[5, 6]. Construct boundaries comprised residues 557-1106 for full-length PDGFRB ($PDGFRB^{FL}$) and residues 584-1106 for truncated PDGFRB. The highest ranked structural model is presented (from a total of five cycles), following AMBER relaxation[7]. The confidence score of the AlphaFold2 model is highlighted using B-factor colouring (Supp. Figure 2b), where final structural figures were generated using ChimeraX[8].

Recombinant protein expression and purification

The intracellular domain of PDGFRB (residues 557-1106), or the truncated, form encoded by the $CD74^{intr}$::PDGFRB fusion form (residues 584-1106), was were amplified from the full-length PDGFRB gene and subcloned into pFastBac Htb (LifeTechnologies) as BamHI-EcoRI fragments, which encodes a TEV protease-cleavable N-terminal hexahistidine tag. Bacmids were prepared in from DH10MultiBac Escherichia coli (ATG Biosynthetics) using established methods[9] and introduced into *Sf*21 cells by Cellfectin II (Thermo Fisher Scientific) mediated

transfection in six-well plates using the Bac-to-Bac protocol (Thermo Fisher Scientific) and Insect-XPRESS (Lonza) media, as detailed previously[9]. P1 virus was harvested after 4 days at 27 °C (static incubation) and added at 1% v/v to 100 mL Sf21 cells at 0.5 × 10^6 density. After 2 days at 27 °C, 120 rpm, P2 virus was harvested. P2 virus (1 mL) was then added to 0.5 L *Sf*21 (1-1.5 × 10^6 cells/mL) in 2.8 L Fernbach flasks, and cells cultured for 48 h at 27 °C, 90 rpm. Cells were harvested via centrifugation at 500 × g and pellets were flash-frozen in liquid nitrogen and stored at −80 °C. Pellets were resuspended in low imidazole buffer [20 mM HEPES (pH 7.5), 200 mM NaCl, 5 mM imidazole, 5% glycerol, 0.5 mM TCEP], supplemented with EDTA-free cOmplete Protease Inhibitor (Roche) and lysed by sonication. Cell debris and insoluble material was pelleted via centrifugation at 45,000 × g and the lysate was incubated with pre-equilibrated His-Tag Ni-NTA resin (Roche) at 4 °C for 1 h with gentle agitation. Ni-NTA beads were then pelleted via centrifugation (500 x g) and washed thoroughly with low imidazole buffer, followed by a buffer with 35 mM imidazole. Bound protein was eluted from the beads using a high imidazole buffer [20 mM HEPES (pH 7.5), 200 mM NaCl, 250 mM imidazole, 5% glycerol, 0.5 mM TCEP], filtered through a 0.45-µm filter, mixed with 300 µg of recombinant His_6-TEV protease and dialysed overnight in size exclusion buffer [20 mM HEPES (pH 7.5), 200 mM NaCl, 5% glycerol] at 4 °C. Following TEV protease cleavage, the dialysate was incubated with Ni-NTA resin (Roche) pre-equilibrated in size exclusion buffer at 4 °C for 1 h to remove His_6-TEV and uncut protein. Following incubation, the supernatant was filtered through a 0.45-µm filter, concentrated via centrifugal ultrafiltration (30 kDa molecular weight cut-off; Millipore) and loaded onto a HiLoad 16/600 Superdex 200 prep grade size exclusion column (Cytiva) equilibrated in size exclusion buffer. Purity was assessed following resolution by reducing StainFree SDS-PAGE gel electrophoresis (BioRad). Protein Pure fractions were pooled, concentrated by centrifugal ultrafiltration, was then aliquoted, flash-frozen in liquid nitrogen and stored at −80 °C.

Analytical size exclusion chromatography

Analytical size exclusion chromatography runs were performed in size exclusion buffer (buffer [20 mM HEPES (pH 7.5), 200 mM NaCl, 5% glycerol] on a Superdex 200 10/300 Increase GL column (Cytiva). Purified protein was injected (50 µL) onto the column at ~3 mg/mL. Gel filtration standard (BioRad) was used to define the molecular weight marker elution volumes (1.35-670 kDa) and the PK-1 kinase dimer (~66kD)[10] included for comparison.

In vitro kinase activity assay

In vitro kinase activity was measured using an ADP-Glo kinase assay kit (Promega), following standard procedures. Briefly, a 10 µL kinase reaction was assembled, containinged 50 ng PDGFRB, 250 ng Poly-(Glu,Tyr 4:1) peptide substrate (Sigma), and 200 µM ATP in kinase buffer [50 mM HEPES (pH 7.5), 20 mM MgCl2, 2 mM MnCl2, 1 mM TCEP]. Each reaction was performed at 30 °C for 30mins and terminated by the addition of the ADP-Glo reagent. After incubation for 40 min with the ADP-Glo reagent, the Kinase Detection reagent was added and luminescence was detected with a microplate reader (CLARIOstar, BMG LabTech) after 30 min incubation.

Complete cDNA sequence and description of CD74^{intr}::PDGFRB mutants

All tags and mutations/deletions were generated using site directed mutagenesis and InFusion cloning (Takara) following manufacturer's instructions. The full *CD74^{intr}::PDGFRB* cDNA amplified from the patient is indicated below, as well as the precise location of each tag and mutant.

1. FLAG and V5 tags

Flag tag: GATTACAAGGATGACGACGATAAG

V5 tag: GGTAAGCCTATCCCTAACCCTCTCCTCGGTCTCGATTCTACG

N-terminal FLAG: inserted after nucleotide 3 (ATG) of CD74::PDGFRB

C-terminal FLAG: inserted after nucleotide 1854 (just prior to stop codon) of CD74::PDGFRB

C-terminal V5: inserted after nucleotide 1854 (just prior to stop codon) of CD74::PDGFRB

2. Mutants

ATG1 del: bp 1_3 del

TTG mut: bp 151-153 TTG>AAA

Partial intron del: bp 126_153 del

Intron del: bp 126_200 del

ATG2 mut: bp 289-291 ATG>GCG

CD74^{intr}::PDGFRB fusion

<CD74>ATGCACAGGAGGAGAAGCAGGAGCTGTCGGGAAGATCAGAAGCCAGTCATGGATGACCA
GCGCGACCTTATCTCCAACAATGAGCAACTGCCCATGCTGGGCCGGCGCCCTGGGGCCCCGGAGA
GGATGGATATTACCATTTGAAATACTTTGGCGCTTGCAGTGAGATCTTGCTTAAAAGAAGAAACCC
ACCAAATTC<PDGFRB>TTTGGCAGAAGAAGCCACGTTACGAGATCCGATGGAAGGTGATTGAGTC
TGTGAGCTCTGACGGCCATGAGTACATCTACGTGGACCCCATGCAGCTGCCCTATGACTCCACGTG
GGAGCTGCCGCGGGACCAGCTTGTGCTGGGACGCACCCTCGGCTCTGGGGCCTTTGGGCAGGTGG

TGGAGGCCACGGCTCATGGCCTGAGCCATTCTCAGGCCACGATGAAAGTGGCCGTCAAGATGCTT
AAATCCACAGCCCGCAGCAGTGAGAAGCAAGCCCTTATGTCGGAGCTGAAGATCATGAGTCACCT
TGGGCCCCACCTGAACGTGGTCAACCTGTTGGGGGCCTGCACCAAAGGAGGACCCATCTATATCAT
CACTGAGTACTGCCGCTACGGAGACCTGGTGGACTACCTGCACCGCAACAAACACACCTTCCTGCA
GCACCACTCCGACAAGCGCCGCCCGCCCAGCGCGGAGCTCTACAGCAATGCTCTGCCCGTTGGGCT
CCCCCTGCCCAGCCATGTGTCCTTGACCGGGGAGAGCGACGGTGGCTACATGGACATGAGCAAGG
ACGAGTCGGTGGACTATGTGCCCATGCTGGACATGAAAGGAGACGTCAAATATGCAGACATCGAG
TCCTCCAACTACATGGCCCCTTACGATAACTACGTTCCCTCTGCCCCTGAGAGGACCTGCCGAGCA
ACTTTGATCAACGAGTCTCCAGTGCTAAGCTACATGGACCTCGTGGGCTTCAGCTACCAGGTGGCC
AATGGCATGGAGTTTCTGGCCTCCAAGAACTGCGTCCACAGAGACCTGGCGGCTAGGAACGTGCT
CATCTGTGAAGGCAAGCTGGTCAAGATCTGTGACTTTGGCCTGGCTCGAGACATCATGCGGGACTC
GAATTACATCTCCAAAGGCAGCACCTTTTTGCCTTTAAAGTGGATGGCTCCGGAGAGCATCTTCAA
CAGCCTCTACACCACCCTGAGCGACGTGTGGTCCTTCGGGATCCTGCTCTGGGAGATCTTCACCTT
GGGTGGCACCCCTTACCCAGAGCTGCCCATGAACGAGCAGTTCTACAATGCCATCAAACGGGGTT
ACCGCATGGCCCAGCCTGCCCATGCCTCCGACGAGATCTATGAGATCATGCAGAAGTGCTGGGAA
GAGAAGTTTGAGATTCGGCCCCCCTTCTCCCAGCTGGTGCTGCTTCTCGAGAGACTGTTGGGCGAA
GGTTACAAAAAGAAGTACCAGCAGGTGGATGAGGAGTTTCTGAGGAGTGACCACCCAGCCATCCT
TCGGTCCCAGGCCCGCTTGCCTGGGTTCCATGGCCTCCGATCTCCCCTGGACACCAGCTCCGTCCTC
TATACTGCCGTGCAGCCCAATGAGGGTGACAACGACTATATCATCCCCCTGCCTGACCCCAAACCC
GAGGTTGCTGACGAGGGCCCACTGGAGGGTTCCCCCAGCCTAGCCAGCTCCACCCTGAATGAAGT
CAACACCTCCTCAACCATCTCCTGTGACAGCCCCCTGGAGCCCCAGGACGAACCAGAGCCAGAGC
CCCAGCTTGAGCTCCAGGTGGAGCCGGAGCCAGAGCTGGAACAGTTGCCGGATTCGGGGTGCCCT
GCGCCTCGGGCGGAAGCAGAGGATAGCTTCCTGTAG

Supplementary Figure Legends

Supplementary Figure 1.

(a) Plot of RNA expression (Transcripts per million) of the indicated genes in a set of paediatric B-ALL patients (EGAD00001006335[11]). The expression of each gene is shown in all samples, with the coloured dots indicating samples with Ph-like B-ALL based on expression profiles and the presence of a driver lesion. The case described in this letter is indicated. **(b)** PCR of the patient with *CD74^{intr}::PDGFRB* using primer sets flanking the predicted breakpoint sequence (lanes 1-2) and full-length transcript (lane 3). A no template control reaction was included (lane 4). **(c)** Left panel: Schematic of methodology used to generate murine pre-B cells. Right panel: Western blot analysis of BCR::ABL1 and CD74^{intr}::PDGFRB expressing primary mouse pre-B cells treated with 0, 1 or 5 µM imatinib for 6 hours. Cells expressing the BCR::ABL1 or CD74^{intr}::PDGFRB were first selected in the absence of IL-7. **(d)** Western blot analysis of Ba/F3 lines expressing an empty vector (EV) control, CD74::ABL1 hybrid containing the ABL1 portion from the BCR::ABL1 p190 fusion, or the equivalent ABL1 domain alone with the addition of a 5' ATG. Diagram depicts expected protein size based on expression of two alternate open reading frames.

Supplementary Figure 2.

(a) Analytical size exclusion chromatography of recombinant PDGFRB$^{557-1106}$ and the truncated form PDGFRB$^{584-1106}$ encoded by the CD74^{intr}::PDGFRB fusion. The elution volumes of molecular weight standards, shown above the chromatograms, support that both constructs are dimeric in solution when compared to the monomeric molecular weight in the SDS-PAGE gel (**Figure 2g**). This is further supported by the elution profile of a model protein, PK-1, with a dimeric molecular weight of 66 kDa (grey dash)[10]. Absorbance at 280 nm (A280nm) is normalized across all samples. **(b)** AlphaFold2 structural modelling of full length PDGFRB (PDGFRB^{FL}) and CD74::PDGFRB highlighting loss of juxtamembrane region in CD74^{intr}::PDGFRB. Confidence of each AlpaFold2 model is indicated on a spectrum from low (blue) to high (red). **(c)** Immunofluorescence of Ba/F3 lines expressing PDGFRB variants. **(d)** Ba/F3 cells containing fusions or empty vector (expressing GFP) were mixed 1:1 with parental cells and analysed by flow cytometry for percentage of GFP following IL-3 withdrawal. Cells were cultured in the presence or absence of 1µM imatinib. Data shown is representative of 3 independent experiments.

References

1. Dobin A, Davis CA, Schlesinger F, Drenkow J, Zaleski C, Jha S, *et al.* STAR: ultrafast universal RNA-seq aligner. *Bioinformatics* 2013 Jan 1; **29**(1)**:** 15-21.

2. Bolger AM, Lohse M, Usadel B. Trimmomatic: a flexible trimmer for Illumina sequence data. *Bioinformatics* 2014 Aug 1; **30**(15)**:** 2114-2120.

3. Uhrig S, Ellermann J, Walther T, Burkhardt P, Frohlich M, Hutter B*, et al.* Accurate and efficient detection of gene fusions from RNA sequencing data. *Genome Res* 2021 Mar; **31**(3)**:** 448-460.

4. Sadras T, Martin M, Kume K, Robinson ME, Saravanakumar S, Lenz G*, et al.* Developmental partitioning of SYK and ZAP70 prevents autoimmunity and cancer. *Mol Cell* 2021 May 20; **81**(10)**:** 2094-2111 e2099.

5. Jumper J, Evans R, Pritzel A, Green T, Figurnov M, Ronneberger O*, et al.* Highly accurate protein structure prediction with AlphaFold. *Nature* 2021 Aug; **596**(7873)**:** 583-589.

6. Mirdita M, Schutze K, Moriwaki Y, Heo L, Ovchinnikov S, Steinegger M. ColabFold: making protein folding accessible to all. *Nat Methods* 2022 Jun; **19**(6)**:** 679-682.

7. Eastman P, Swails J, Chodera JD, McGibbon RT, Zhao Y, Beauchamp KA*, et al.* OpenMM 7: Rapid development of high performance algorithms for molecular dynamics. *PLoS Comput Biol* 2017 Jul; **13**(7)**:** e1005659.

8. Pettersen EF, Goddard TD, Huang CC, Meng EC, Couch GS, Croll TI*, et al.* UCSF ChimeraX: Structure visualization for researchers, educators, and developers. *Protein Sci* 2021 Jan; **30**(1)**:** 70-82.

9. Fitzgibbon C, Meng Y, Murphy JM. Co-expression of recombinant RIPK3:MLKL complexes using the baculovirus-insect cell system. *Methods Enzymol* 2022; **667:** 183-227.

10. Oliver MR, Horne CR, Shrestha S, Keown JR, Liang LY, Young SN*, et al.* Granulovirus PK-1 kinase activity relies on a side-to-side dimerization mode centered on the regulatory alphaC helix. *Nat Commun* 2021 Feb 12; **12**(1)**:** 1002.

11. Brown LM, Lonsdale A, Zhu A, Davidson NM, Schmidt B, Hawkins A*, et al.* The application of RNA sequencing for the diagnosis and genomic classification of pediatric acute lymphoblastic leukemia. *Blood Adv* 2020 Mar 10; **4**(5)**:** 930-942.

Supplementary Figure 1.

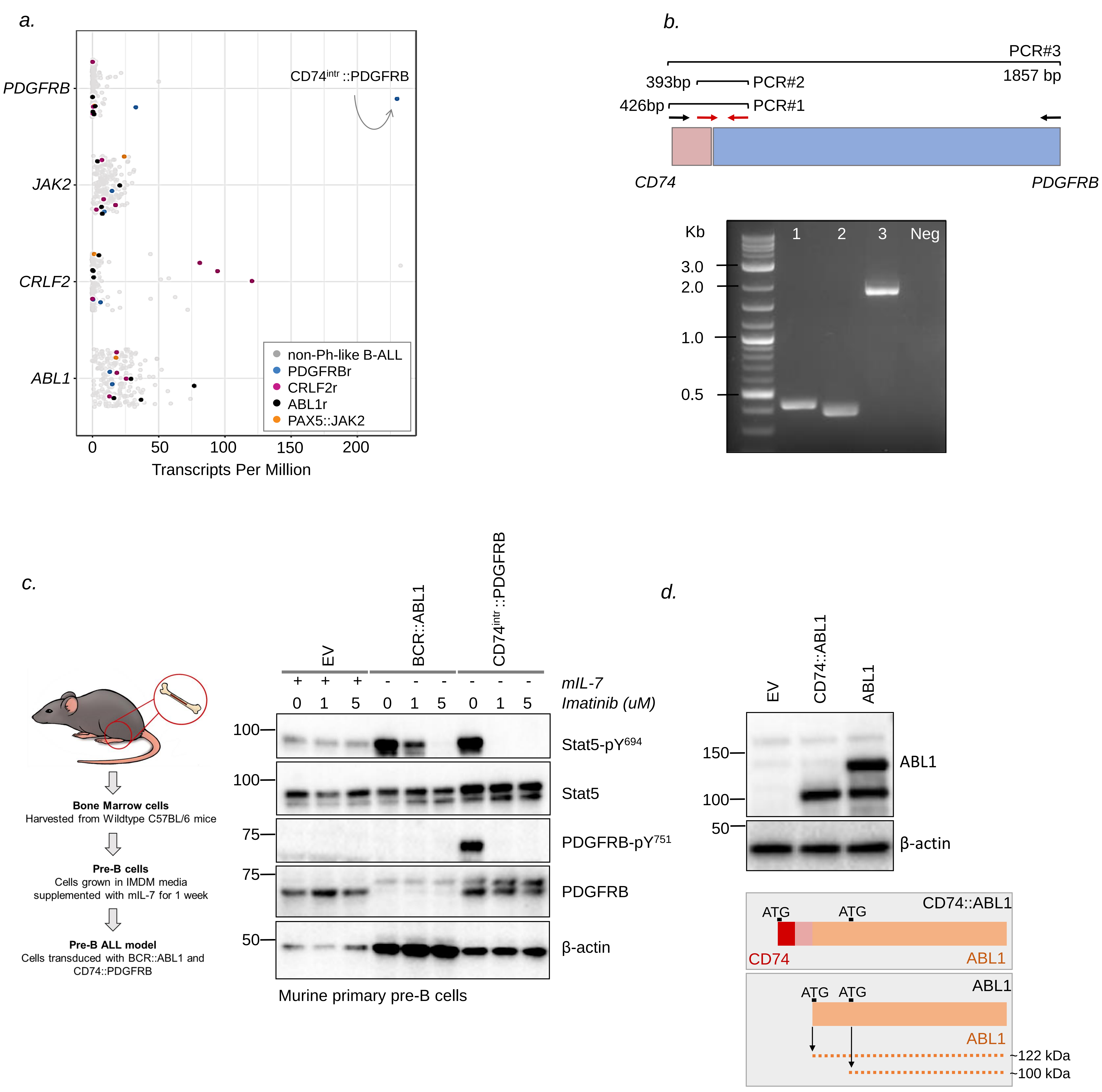

Supplementary Figure 2.

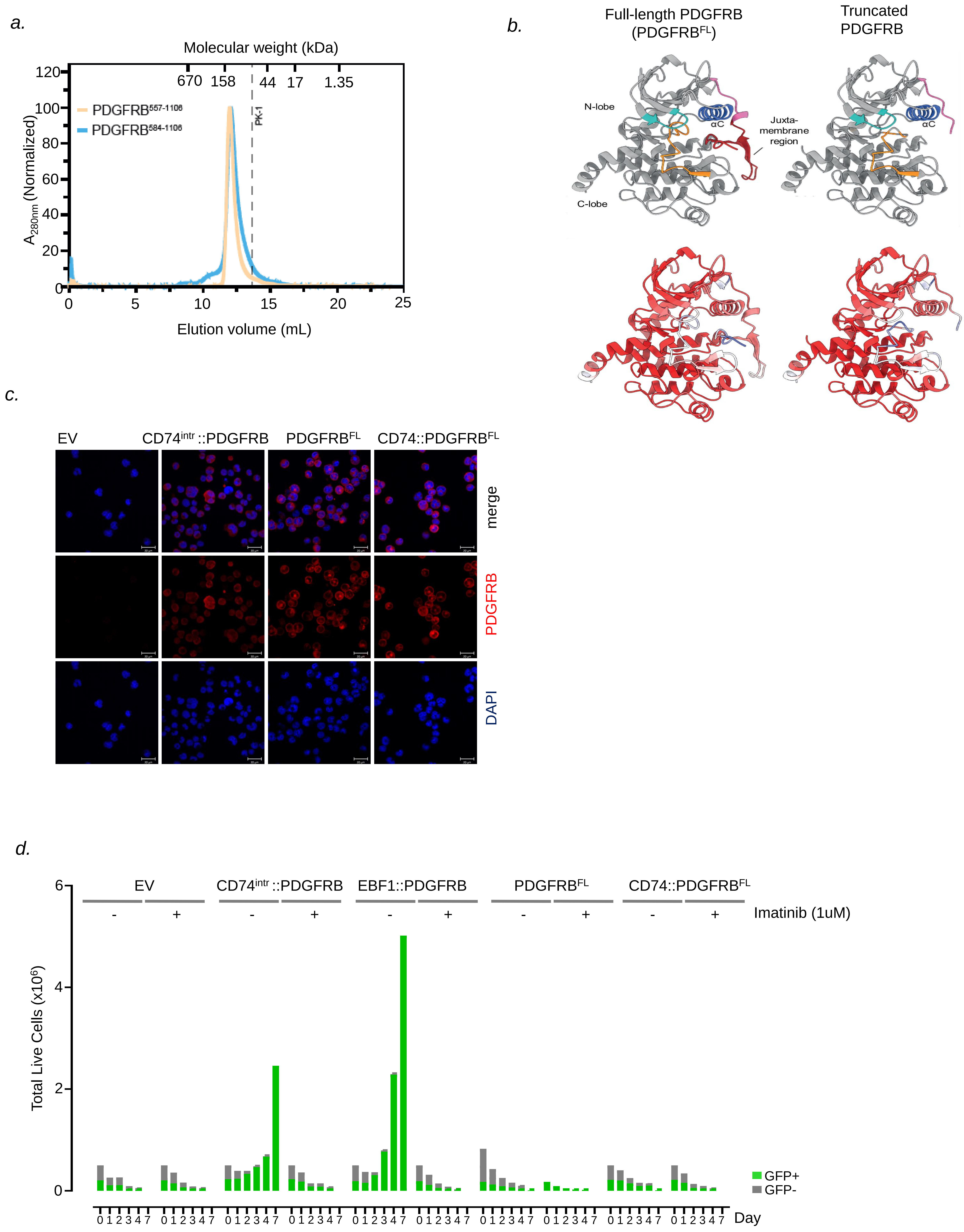

Supplement: Supplementary file 1 — Supplementary Materials, Methods and Figures [file 41375_2023_1843_MOESM1_ESM.pdf]
